# Social and locomotor play behavior of dairy calves kept with the dam either full time or half time in straw-bedded pens

**DOI:** 10.3168/jdsc.2022-0337

**Published:** 2023-04-18

**Authors:** E. Bailly-Caumette, M. Bertelsen, M.B. Jensen

**Affiliations:** 1 Department of Biology, Ecole Normale Supérieure de Lyon, 69007 Lyon, France; 2 Department of Animal and Veterinary Sciences, Aarhus University, 8830 Tjele, Denmark

## Abstract

This study investigated play behavior of dairy calves kept with the cow either full time or half time in groups of 4 dam-calf pairs in 68-m^2^ straw-bedded pens. Twenty-four of 48 calves entering the experiment (2 calves in each of 12 pens) were observed during 24 h at 3 and 7 wk of age. The total duration of locomotor play did not differ between the 2 treatments, but full-time calves performed less frontal pushing (social play). Irrespective of treatment, calves performed less parallel locomotor play and more frontal pushing at 7 wk than 3 wk of age. Analyzing the intensity of play behavior during milking times (morning and afternoon, respectively), during the night and between morning and afternoon milking, showed that calves performed locomotor play more intensively after the cows had left the pen for milking than during other periods. The results illustrate the importance of space and external stimulation for the performance of play behavior.

## Highlights

• The duration of locomotor play did not differ between full- and half-time calves.
• Half-time calves performed more frontal pushing than full-time calves.
• Calves performed locomotor play more intensively after the cows had left the pen for milking than during other periods.

## Graphical Abstract

**Figure:**
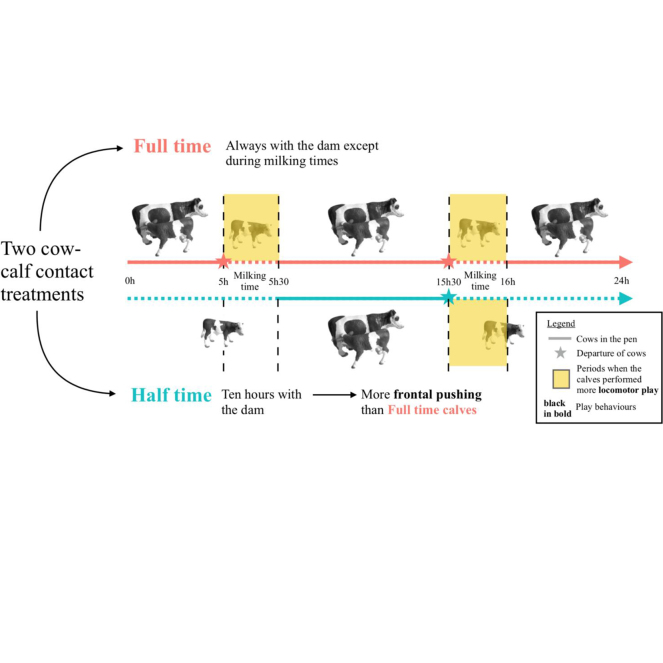
**Summary:** The study investigated play behavior of dairy calves kept with the dam either full time or half time. The total duration of play behavior did not differ between treatments, but full-time calves performed less frontal pushing and tended to perform less synchronous locomotor play, indicating that elements of play requiring the most space were reduced in full-time calves. The intensity of locomotor play behavior was higher after the cows had left the pen to go to milking (i.e., during the 2 milking times for full-time calves and during the afternoon milking for half-time calves) compared with other times of day. This indicates that the sudden availability of a larger space stimulated play behavior. The duration of locomotor play was similar in weeks 3 and 7, while the duration of social play increased.

On most modern dairy farms, the calf is routinely separated from the dam within 24 h after birth (Bolton and von Keyserlingk, 2021). However, early separation of cow and calf deprives the calf of maternal care, which is found to affect its social competences negatively (Flower and Weary, 2001; Meagher et al., 2019). This practice is therefore questionable from an animal welfare point of view and is increasingly criticized (Busch et al., 2017). Nevertheless, other practices are possible such as various cow-calf contact systems. For instance, full-time cow-calf contact means that the calf is always with the dam except during milking, and half-time contact implies that the calf is with the dam either only during the day or during the night (Sirovnik et al., 2020). There is an increasing interest in dairy cow-calf contact systems, but as current dairy cow housing is not suitable for calves, half-time contact may be a more feasible cow-calf system than full-time contact (Johnsen et al., 2016). However, it remains to be investigated how part-time cow-calf contact compares with full-time contact with regard to impacts on calf welfare (Meagher et al., 2019).

It is increasingly recognized that good animal welfare requires the presence of positive emotions, and knowing how to assess these is a crucial (Boissy et al., 2007) but challenging task (Désiré et al., 2002). One promising positive welfare indicator in juveniles is play behavior given its motivational basis (Boissy et al., 2007). First, the juvenile is motivated to play when it is in a relaxed or low stress condition with no threats to welfare, such as hunger, cold, or fear, and second, the performance of play is pleasurable (Burghardt, 2005). Thus, the presence of play behavior may indicate a state of positive welfare, meaning that not only are the primary needs met, but the animals are also experiencing a positive affective state (Boissy et al., 2007). However, the interpretation of play behavior as a welfare indicator is complicated. For instance, locomotor play is subject to rebound after a period of nonperformance and may thus not consistently reflect favorable environmental conditions (Held and Spinka, 2011).

In calves, play behaviors are mainly locomotor play and social play (Reinhardt, 1980). Locomotor play can be performed alone (individual locomotor play) or with other calves at the same time (parallel locomotor play) and includes jumping, hind leg kicks, and galloping (Jensen et al., 1998). Locomotor play was performed more when more space was available (Jensen et al., 1998; Jensen and Kyhn, 2000), and more calves also performed more locomotor play at the same time when space was increased from 1.5 to 4 m^2^ per calf (Jensen and Kyhn, 2000). Social play includes reciprocal frontal pushing of another calf's head or neck (Jensen et al., 1998) and has been observed between calf and dam a few days after birth (Jensen, 2011). This type of play behavior increased with age in gazelles (Gomendio, 1988), while locomotor play peaked early on and then declined to low values, especially in low-fed and early-weaned calves (Krachun et al., 2010).

The aim of this study was to investigate the effect of full- versus half-time cow-calf contact on the duration and timing of locomotor and social play behavior at 2 different calf ages. We hypothesized (1) that full-time calves perform more play behavior than half-time calves due to the higher level of maternal contact, (2) that calves play the most when cows leave the pen, and (3) that calves perform more social play and less locomotor play as they grow older.

The study was conducted from September 2020 to April 2021 in the experimental barn at the Danish Cattle Research Centre at Foulum, Aarhus University, Denmark. All procedures involving animals were in accordance with Danish law and the Danish Animal Experiments Inspectorate regulations. Forty-eight purebred Danish Holstein cows and their newborn calves were allocated to 6 blocks according to calving date. In each block, the cow-calf pairs were allocated to 2 groups of 4 pairs (full-time or half-time contact) balancing for dam parity and calf sex. To be enrolled in the experiment, cows had to have calved without complications or assistance, and both cow and calf had to be clinically healthy. Three cow-calf pairs were not included because the cow was aggressive toward the calf, or because the calf did not suck without assistance within 24 h of birth. Cows were in first to fifth lactation, and at least 1 first-lactation cow was included per group. None of the cows had previously been kept with their calf for longer than 24 h after birth. The age difference between the youngest and oldest calf in a block was on average 13 d (SD = 6.42 d), ranging from 4 to 24 d.

After calving, the pair stayed in the individual calving pen for at least 24 h, and suckling was assisted if needed. The pair was then moved to one of the deep-bedded experimental group pens (9 m × 7.5 m) for 4 cow-calf pairs on the same treatment. A feed trough and 2 water bowls were positioned in the front of the pen. In the back of the pen, 2 calf creeps positioned in each corner (3 m × 3 m and 1.5 m × 1.5 m, respectively) were accessible to calves only. In both calf creeps, calves had access to hay and concentrates ad libitum, whereas only the largest calf creep contained a water bowl. Calves also had access to the cows' TMR. The TMR and straw bedding were added every day in the morning at approximately 0845 h. Concentrate and hay were added as needed.

All cows were milked between 0500 and 0530 h and between 1530 and 1600 h. Full-time cows were only separated from their calves for milking (i.e., 2 × 30 min/d). Half-time cows were moved to a separation pen (concrete floor, 1 cubicle per cow, ad libitum access to water, and the same TMR as in the experimental pens) after the afternoon milking and remained there until the morning milking where they returned to the experimental pens with their calves. Half-time calves did not have access to an alternative milk source while the cows were away from the pen.

Calves were observed at age of 22 (SD = 7) d (range from 3 to 36 d; wk 3) and 50 (SD = 8) d (range from 27 to 66 d; wk 7) of age. Two calves were randomly selected per pen. In 5 pens (2 full-time and 3 half-time pens) both a bull and a heifer were observed, in 4 pens both observed calves were heifers (3 full-time and 1 half-time pens), and in 3 pens both observed calves were bulls (1 full-time and 2 half-time pens). In total, 4 bulls and 8 heifers were observed in the full-time treatment, and 7 bulls and 5 heifers were observed in the half-time treatment. The videos were recorded by cameras placed above each pen and stored on hard drives. The behavior of the calves was recorded continuously during 24 h (starting at midnight) by focal animal sampling (Bateson and Martin, 2021) by one observer (the first author) using the Boris software (Friard and Gamba, 2016). Play behavior was recorded following the ethogram by Jensen et al. (1998) and included “locomotor play” (gallop, leap, jump, buck, buck-kick, turn, or body or head twists) scored as either “individual locomotor play” (performed by only the focal calf at the time) or “parallel locomotor play“ (performed simultaneously with at least 1 other calf in the pen). Play behavior also included “frontal pushing“ [the focal calf standing front to front with another individual while the two are mutually pushing their foreheads against each other without resulting in withdrawal or aggressive behavior (whether the reactor was a calf, the dam, or an alien cow was recorded)] and “object play“ (pushing forehead against pen fixtures or straw, or rubbing head and neck in straw bedding while kneeling). In addition, “head butting” was observed [pushing forehead against the neck or body of another individual without resulting in withdrawal or aggressive behavior (whether the reactor was a calf, the dam, or an alien cow was recorded)]. Finally, the exact times of departure from and return to the experimental pens of the cows were noted for each block and day, as well as the duration of any human presence in the pen. All recordings were done by the same observer, and to test the intraobserver reliability the first video was watched a second time after all data were collected, and the 2 observations were compared using Cohen's kappa (κ = 0.932).

Analysis was conducted in SAS (PROC MIXED; SAS version 9.4, SAS Institute Inc.). Figures were produced using the package ggplot2 (Wickham, 2011). In wk 7, 3 heifer calves and their dam had to be removed from the experiment for health issues (blocks 3, 4, and 6), and thus there were only 9 full-time calves observed in wk 7. For each calf and observation day, the duration in seconds was calculated for the following variables: (1) individual locomotor play, (2) parallel locomotor play, (3) frontal pushing with another calf), (4) head butting another calf, (5) head butting the dam, and (6) object play. In addition, total duration of all play behaviors (as defined above) and the total duration of locomotor play (individual and parallel) were calculated per calf and observation day. Then the mean over the 2 calves in the same pen was calculated per week and these variables were analyzed using a linear mixed model including the fixed effects treatment (full time or half time), week (3 or 7), and the 2-way interaction between treatment and week. The pen was included as a random effect to account for the dependence between repeated observations of the same pens. The assumptions of normal distribution and homoscedasticity were checked by graphical inspection of the residuals. Less than 50% of the calves performed frontal pushing of the dam and head butting toward an alien cow, and these variables were transformed into binary variables. The number of calves performing frontal pushing of the dam and the number of calves head butting an alien cow were analyzed using Fisher's exact test by week. Finally, we assessed the impact of the daily absence of the cows on the distribution of play behaviors (locomotor play and frontal pushing) during the 24 h. Four periods of day were defined according to the milking times: first milking (from the departure of the full-time cows to the first milking to the return of all cows in the pen, approximately 30 min), between milking times (from the return of the cows from the first milking to their departure to the second milking, approximately 10 h), second milking (from the departure of the cows to the second milking to the return of full-time cows, approximately 30 min) and night (from the return of full-time cows from the second milking to their departure to the first milking in the morning, approximately 13 h). For each of the 4 periods, the duration of locomotor play and the duration of frontal pushing were divided by the duration of this period (all in s) and multiplied by 3,600 to calculate the hourly rate. A mean of the 2 calves in each pen was taken within week. These data were analyzed by a linear mixed model including the fixed effects of treatment, period, and week, and the interaction between treatment and period, as well as treatment and week. The pen was included as random effects to account for the 2 observations on each pen.

The total duration of all play behaviors, the total duration of locomotor play, and the duration of individual locomotor play did not differ between the 2 treatments (Table 1). However, full-time calves tended to perform less locomotor play in parallel with other calves than half-time calves. Regarding social play behavior, full-time calves performed less frontal pushing of another calf, but tended to perform more head butting of another calf, than half-time calves. There was no difference between the 2 treatments regarding the duration of object play. Nor were there any difference regarding the number of calves frontal pushing the dam or head butting an alien cow. In wk 3, 42% of the calves performed frontal pushing with the dam and 38% of the calves performed head butting with an alien cow, whereas in wk 7, the corresponding values were 39% and 62%. Frontal pushing was never performed with an alien cow.

**Table 1:** Duration (s/24 h) of play behaviors [LSM and 95% confidence limits (CL)] of calves with full-time and half-time contact with their dam

Item	Full time		Half time		*F*_1,10_	*P*-value
	Mean	95% CL	Mean	95% CL		
Total duration of play behavior	631	407–855	673	449–897	0.09	0.775
Locomotor play						
Total	168	114–222	192	138–246	0.48	0.505
Individual	134	96–172	123	85–161	0.22	0.648
Parallel	34.1	4.87–63.3	69.2	40.0–98.5	3.58	0.088
Frontal pushing						
Frontal pushing calf	85.2	12.2–158	199	126–272	6.06	0.034
Head butting						
Calf	150	92.7–207	83	26.0–141	3.37	0.0964
Dam	152	81.8–222	78.8	8.63–149	2.70	0.1312
Object play	28.4	14.6–42.1	40.1	26.5–53.8	1.81	0.2088

Calves performed less parallel locomotor play in wk 7 than in wk 3 (Table 2). However, calves performed more frontal pushing of another calf and tended to perform more head butting of another calf and more head butting of the dam in wk 7 than in wk 3. Finally, calves performed less object play in wk 7 than wk 3.

**Table 2:** Duration (s/24 h) of play behaviors [LSM and 95% confidence limits (CL)] of calves in wk 3 and 7

Item	wk 3		wk 7		*F*_1,10_	*P*-value
	Mean	95% CL	Mean	95% CL		
Total duration of play behavior	549	351–748	755	556–953	3.65	0.085
Locomotor play						
Total	200	137–264	160	96.2–224	0.78	0.399
Individual	127	81.1–173	130	83.5–176	0.00	0.945
Parallel	73.0	45.6–100	30.3	2.94–57.8	6.98	0.025
Frontal pushing						
Frontal pushing calf	102	38.6–165	183	119–246	5.97	0.035
Head butting						
Calf	77.1	15.0–139	156	94.1–218	3.52	0.090
Dam	75.8	10.3–141	155	89.3–220	4.23	0.067
Object play	49.1	32.8–65.4	19.4	3.10–35.7	6.42	0.030

The hourly rate of all locomotor play behavior showed a treatment × period interaction (*F*_3,58.2_ = 4.76; *P* = 0.005). Full-time calves performed locomotor play more intensively during the 2 milking times than between milking times and during the night (with no difference between the 2 milking times), whereas half-time calves performed locomotor play more intensively during the afternoon milking time than during the other periods (Figure 1). Comparing the 2 treatments within each of the 2 milking times, full-time calves performed locomotor play more intensively during the morning milking time, but less intensively during the afternoon milking, compared with half-time calves. For frontal pushing no interaction between treatment and period was found (*F*_3,58.8_ = 1.68; *P* = 0.181), but the effect of period was significant (*F*_3,58.2_ = 7.95; *P* < 0.001) due to a higher intensity of frontal pushing during afternoon milking time than during any of the other 3 periods [3.28, 2.0, 6.18, 2.0 (SE 0.867), for morning, between, afternoon, and night (square root transformed); back transformed mean estimates were 10.8, 4.0, 38.2, and 4.0 s/h].

**Figure 1:**
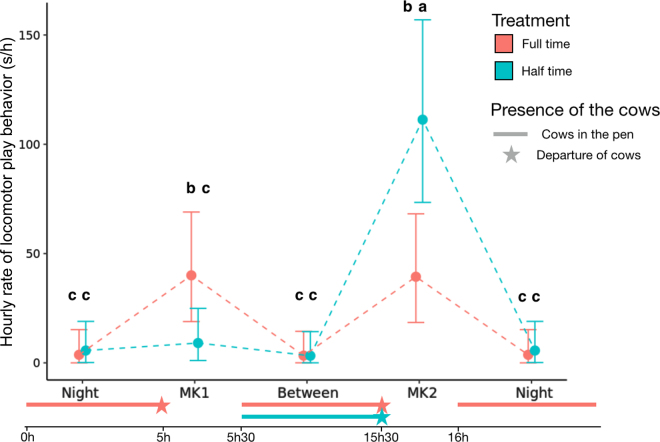
Comparison of hourly rate of locomotor play between the different periods of the day for full-time and half-time calves. The periods of the day concerned are the night, the first milking time (MK1), between the 2 milking times (Between), and the second milking time (MK2). The segments represent the periods of the day when the cows are in the pen. Full-time calves are shown in orange and half-time calves in blue. Estimates with different letters (a–c) are significantly different (*P* < 0.05). Error bars represent 95% CI.

We aimed to investigate the effect of full- versus half-time dam contact performance of play behavior. We first hypothesized that full-time calves would perform more play behavior than half-time calves due to longer maternal contact time. Contrary to this hypothesis, the total duration of play behavior was not different between the 2 groups. However, considering the different types of play behaviors, full-time calves performed less frontal pushing (social play), and tended to perform less parallel locomotor play and more head butting.

Frontal pushing and parallel locomotor play are both play behaviors that involve the simultaneous engagement of at least 2 calves. Somers (2012) found that calves maintaining a front-to-front position, while performing frontal pushing, took up 3.5 m^2^ of free space, while galloping (a prominent element of locomotor play behavior) was performed on an average distance of 13.5 m. Although frontal pushing is more clearly distinguishable social play, we also consider head butting (i.e., in a nonagonistic context as defined here) a social play behavior. As head butting requires less space than frontal pushing, it may be performed more when the space is limiting performing frontal pushing. Therefore, one explanation of full-time calves performing less frontal pushing and tending to perform both less parallel locomotor play and more head butting may be that they have less space available on a daily basis because cows were in the pen for most of the 24 h.

When we analyzed the diurnal distribution of play, the intensity of all locomotor play behavior was higher after the cows had left the pen to go to milking (i.e., during the 2 milking times for full-time calves and during the afternoon milking for half-time calves) compared with other times of day. This finding is in support of our second hypothesis, namely that calves played the most when the cows had left the pen. Indeed, the sudden increase in space available may have stimulated locomotor play in a similar way as release in a larger area stimulated locomotor play behavior (Jensen, 1999). However, the sudden increase in space was confounded with the departure of the cows, which could also act as an external stimulation of play, similar to adding straw to the pen (Jensen et al., 1998; Duve et al., 2012). Nevertheless, these interpretations do not explain why frontal pushing showed similar distributions over the periods of day on the 2 treatments and we encourage future studies to clarify the effect of space and external stimulation, respectively, on play behavior.

The main limitation of this study is that space available and maternal contact are confounded. Thus, an alternative explanation for more frontal pushing among calves on the half-time treatment may be the reduced maternal contact. Toinon et al. (2022) found that artificially reared goat kids performed more frontal pushing than dam-reared even if they had less space available and suggest that this may be due to less inhibition by the adults. Finally, frontal pushing may provide calves with information of own and others' physical strength and potential competitive abilities (Reinhardt and Reinhardt, 1982). Social play is not associated with aggression, but we cannot rule out that calves use information on competitive abilities to resolve competitive situations. Half-time calves were without any source of milk during the night and may have competed for access to suckle when the cows returned from morning milking. That the increased level of frontal pushing is related to this potential competition cannot be ruled out. The absence of milk during the night may also render half-time calves hungry, which could explain the lower level of locomotor play during morning milking time. Further studies are needed to clarify these aspects.

In accordance with Reinhardt and Reinhardt (1982), frontal pushing was mainly observed between calves, but if it involved an adult, this adult was the calf's dam. Frontal pushing may be related to the development of social competences and the formation of dominance relationships (Reinhardt and Reinhardt, 1982), and we encourage future studies on the influence of the dam on social play behavior.

Third, we hypothesized that calves perform more social play and less locomotor play as they grew older. In accordance with this hypothesis, calves performed more frontal pushing at 7 than at 3 wk. However, the duration of all locomotor play did not decline over week; only the parallel locomotor play declined from wk 3 to 7. Previous studies using a similar ethogram found a decline in locomotor play within a similar age range (Jensen and Kyhn, 2000; Miguel-Pacheco et al., 2015), but this was confounded with a decline in milk allowance, which is known to reduce locomotor play behavior (Krachun et al., 2010), and the lack of decline in locomotor play in the present study may be due to dam-reared calves consuming high levels of milk. However, the decline of parallel locomotor play may be due to calves growing larger and requiring more space to play simultaneously. Finally, as they got older, calves performed less object play including pushing pen fixtures with the forehead, possibly because they performed more frontal pushing with another calf. Further studies are needed to explore the development and function of object play behavior in cattle.

The level of play recorded in this study was generally higher than what has been reported previously using similar ethograms, but with less space and without maternal contact. We observed 225 s of frontal pushing and 160 s of locomotor play per 24 h at 7 wk of age. In other studies, 7-wk-old calves housed in groups of 4 calves offered a milk ration of 3 L/d and with 1.5 to 4 m^2^ per calf performed approximately 170 s of frontal pushing and 30 s of locomotor play per 24 h (Jensen and Kyhn, 2000) and calves housed in groups of 8 calves offered a milk ration of 12 L/d and with 4 m^2^ per calf performed approximately 50 s per 15 h of locomotor play (Miguel-Pacheco et al., 2015). A recent study comparing play behavior in artificially and dam-reared calves found more locomotor play among dam-reared calves and that most locomotor play was conducted in the 25 m long alleys (Waiblinger et al., 2020). Similarly, for adult cows, the provision of an outdoor environment lead to more locomotor behavior compared with cubicle housing (Shepley et al., 2020), and adult cows performed more locomotor behavior when more space was available (Telezhenko et al., 2012). Thus, we suggest that the higher levels of locomotor and social play in our study is best explained by the larger space allowance. Indeed, the calves in the present study were housed in large pens, and subtracting the calf creeps, there were 56 m^2^, or 14 m^2^ per cow-calf pair. Dam contact has also been suggested to stimulate play behavior (Boissy et al., 2007). However, Duve et al. (2012) found that calves housed with only their dam in a 20-m^2^ pen played at similar levels as ad libitum-fed calves housed in 14-m^2^ pens either individually or in pairs, suggesting that the available space is the key factor. There is a gap in the scientific literature on the effect of space allowance between approximately 4 and 14 m^2^ per calf, and we encourage future studies on the effect of space allowances on locomotor and social play behavior.

Play behavior is considered as a promising indicator of animal welfare. However, the different types of play behavior (locomotor, social, and object play) should be studied separately as they may have different motivational basis. Moreover, more studies are needed to clarify the link between these play behaviors and animal welfare. The importance of space versus maternal contact for animal welfare also needs to be clarified.
